# A novel PANoptosis-related long non-coding RNA index to predict prognosis, immune microenvironment and personalised treatment in hepatocellular carcinoma

**DOI:** 10.18632/aging.205488

**Published:** 2024-01-26

**Authors:** Liangliang Wang, Peng Wan, Zhengyang Xu

**Affiliations:** 1 Department of Chemoradiotherapy, The Affiliated People’s Hospital of Ningbo University, Ningbo, China; 2 Chemoradiotherapy Center of Oncology, The Affiliated People’s Hospital of Ningbo University, Ningbo, China

**Keywords:** hepatocellular carcinoma, PANoptosis, lncRNA, prognosis, tumour immune microenvironment

## Abstract

Background: PANoptosis is involved in the interaction of apoptosis, necroptosis and pyroptosis, playing a role in programmed cell death. Moreover, long non-coding RNAs (lncRNAs) regulate the PCD. This work aims to explore the role of PANoptosis-associated lncRNAs in hepatocellular carcinoma (HCC).

Methods: Co-expression analysis identified PANoptosis-associated lncRNAs in HCC. Cox and Least Absolute Shrinkage and Selection Operator (LASSO) algorithms were utilised to filter lncRNAs and establish a PANoptosis-related lncRNA index (PANRI). Additionally, Cox, Kaplan–Meier and receiver operating characteristic (ROC) curves were utilised to systematically evaluate the PANRI. Furthermore, Estimation of STromal and Immune cells in MAlignant Tumor tissues using Expression data (ESTIMATE), single sample gene set enrichment analysis (ssGSEA) and immune checkpoints were performed to analyse the potential of the PANRI in differentiating different tumour immune microenvironment (TIME) populations. The consensus clustering algorithm was used to distinguish individuals with HCC having different TIME subtypes. Finally, HCC cell lines HepG2 were utilised for further validation in *in vitro* experiments.

Results: The PANRI differentiates patients according to risk. Notably, ESTIMATE and ssGSEA algorithms revealed a high immune infiltration status in high-risk patients. Additionally, consensus clustering divided the patients into three clusters to identify different subtypes of TIME. Moreover, i*n vitro* results showed that siRNA-mediated silencing of AL049840.4 inhibited the viability and migration of HepG2 cells and promoted apoptosis.

Conclusions: This is the first PANoptosis-related, lncRNA-based risk index in HCC to assess patient prognosis, TIME and response to immunotherapy. This study offers novel perspectives on the role of PANoptosis-associated lncRNAs in HCC.

## INTRODUCTION

Primary liver cancer is one of the most prevalent malignancies worldwide. The International Agency for Research on Cancer in 2021 reported that liver cancer is the 6th most prevalent malignancy worldwide and has the 3rd highest mortality rate [1]. Hepatocellular carcinoma (HCC), as the most common primary liver cancer type, accounts for 75–85% of all liver cancer types [2, 3]. Despite the rapid advancement of systemic therapy for HCC in the last decade, the emergence of new immunotherapeutic agents and targeted drugs and the innovation of new treatment concepts and protocols, HCC long-term survival rates remain low, apart from the survival of certain individuals to a certain extent [4, 5]. Therefore, the exploration of effective biomarkers for predicting the prognosis of HCC will not only help to elucidate the molecular mechanisms of HCC progression but also help to differentiate patients into different subgroups and determine their clinical outcomes, thus guiding clinical individualised treatment delivery and improving patient outcomes of HCC.

Programmed cell death (PCD) is essential for regulating cell biological behaviours such as cell function and the cell cycle [6–8]. PANoptosis is a novel form of PCD that has been recently demonstrated in 2019. It is involved in an interactive network of apoptosis, necroptosis and pyroptosis and cannot be defined by a single PCD pathway alone [9]. PANoptosis is regulated by the multimeric complex PANoptosome, which consists of upstream signalling molecules and receptors and serves as the ‘master switch’ for inducing PANoptosis [10]. An increasing number of studies have reported a role for PANoptosis in the malignant biological behaviour and evolution of cancer and on the influence on tumour responses to immunotherapy [11–13]. Additionally, recent studies have demonstrated that PANoptosis-based molecular subtyping is helpful in determining the prognosis and therapeutic response of HCC [14, 15], providing a basis for individualised precision therapy [16]. Hence, the exploration of the molecular mechanisms of PANoptosis in HCC has implications for broadening the diagnostic and therapeutic options presented for HCC.

Long non-coding RNAs (lncRNAs) are transcribed RNA products that can influence the malignant biology of tumours by regulating gene expression. They are also useful as prognostic and diagnostic biomarkers for tumours [17–19]. Recent studies have confirmed that PCD-associated lncRNAs influence tumour evolution and the immune microenvironment and are associated with immunotherapeutic responses [20–22]. Additionally, circulating lncRNAs are stably present in plasma and serum and can serve as promising biomarkers [23, 24]. Prognostic models based on lncRNA have also been suggested to have favourable prognostic predictive potential in HCC [25, 26]. Furthermore, biomarkers based on PCD-associated lncRNAs are considered promising in HCC [27–29]. However, the potential of PANoptosis-associated lncRNAs as biomarkers of HCC remains unexplored.

In this study, a PANoptosis-related lncRNA index (PANRI) was developed and the value of PANRI was determined by comprehensively analysing the clinical outcomes, clinicopathological features, tumour immune microenvironment (TIME), tumour mutation burden (TMB), immunotherapy response and individualised treatment in patients with HCC. The PANRI constructed herein can be used as a new biomarker to predict the prognosis and determine the TIME features in HCC, thereby providing a basis for the selection of precise treatment regimens for patients.

## MATERIALS AND METHODS

### Data sources

RNA sequencing (RNA-seq) data for individuals with HCC were obtained from The Cancer Genome Atlas (TCGA)-LIHC cohort (https://portal.gdc.cancer.gov/repository) along with the mutation data and associated clinical information. Inclusion criteria for study individuals were patients who contained both RNA-seq data and information on survival time and survival status. Strawberry Perl version 5.32.1.1 was used to differentiate and extract the mRNA and lncRNA matrices for the TCGA-LIHC cohort. Based on previous studies, 24 PANoptosis-related genes (PRGs) were included in this study [9, 10, 12, 13, 30–36] (Supplementary Table 1). The present work is based on open-source data and is free from ethical approval.

### Identification of PANoptosis-related lncRNAs in HCC

The R package ‘limma’ was utilised to extract the mRNA expression matrix of PRGs from individuals in the TCGA-LIHC cohort. Using co-expression analysis, lncRNAs associated with PRG mRNAs were obtained and identified as PANoptosis-related lncRNAs (Pearson coefficient > 0.4, *p* < 0.001). The packages ‘ggplot2’ and ‘galluvial’ were utilised to construct a Sankey diagram of the network of PANoptosis-associated lncRNAs and genes. Additionally, ‘limma’ was utilised to extract differentially expressed PANoptosis-associated lncRNAs in the TCGA-LIHC cohort (|Log2 fold change| > 1, FDR < 0.05), which were visualised using the package ‘pheatmap’.

### Identification of a PANRI in HCC

All individuals in the TCGA-LIHC cohort were further randomised into training and validation cohorts in a 1:1 ratio. The univariate Cox (uni-Cox) regression algorithm identified PANoptosis-related lncRNAs associated with survival (*p* < 0.05) and the packages ‘survival’ and ‘pheatmap’ were utilised to draw forest and heat maps. The optimal PANoptosis-related lncRNAs for constructing PANRI were selected using the Least Absolute Shrinkage and Selection Operator (LASSO) algorithm. In this process, ‘caret’ was used to solve data training for classification and regression problems, and the ‘glmnet package’ was used for variable selection for PANRI. Furthermore, the packages ‘ggExtra’, ‘tidyverse’ and ‘ggplot2’ were utilised to analyse the correlation between the expression of lncRNAs and PRGs in the PANRI and plot correlation heat maps. The risk score for individuals with HCC was calculated using the following equation:


$$\text{Risk}\;\text{score}=\sum_{i=1}^{n}\;\text{Coefficient}\;(i)\;\times \;\text{Expression}\;(i),$$

where Coefficient represents the regression coefficient of the lncRNA used to develop the PANRI. Accordingly, all samples were classified into high- and low-risk subgroups according to the median score of the training cohort.

### Validation of the PANRI in HCC

To validate the potential of the developed PANRI, the ‘survminer’ and ‘survivor’ were utilised to plot Kaplan–Meier (K-M) curves and assess survival differences between high- and low-risk subgroups in the training, testing and TCGA-LIHC cohorts, respectively. The ‘pheatmap’ was also used to validate the risk index by plotting risk heat maps, risk distribution and survival status maps for the different cohorts. Moreover, the uni-Cox and multivariate Cox (multi-Cox) algorithms were used to determine the ability of the index to independently predict the clinical outcomes of individuals with HCC. The receiver operating characteristic (ROC) curves were utilised to assess the prognostic predictive value of the index, which was achieved using the ‘timeROC’, ‘survminer’ and ‘survival’ packages. Finally, the differences in the expression of the 24 PRGs in the two risk subgroups were further analysed.

### Establishment and evaluation of a nomogram in HCC based on the PANRI

To better evaluate the clinical outcomes of individuals with HCC, we combined the index with clinicopathological features to construct nomograms according to the outcomes of multi-Cox to predict the survival rates of individuals at 1-, 3- and 5-year intervals. Furthermore, the Hosmer–Lemeshow test calibration curves were utilised to assess the predictability and reliability of the nomogram using the ‘rms’, ‘regplot’ and ‘survivor’.

### Validation of the PANRI in clinicopathological parameters

To determine the applicability of the constructed risk index to patients with HCC having different clinicopathological parameters, the ‘survminer’ and ‘survival’ packages were utilised to map the K-M curves of the two risk groups in different clinical subgroups (age, gender, tumour grade and stage). Additionally, ‘ComplexHeatmap’ was utilised to visualise the status of different clinical parameters in the two risk subgroups.

### Functional enrichment analysis of the PANRI

Gene ontology (GO) involves the analysis of three aspects: cellular composition, molecular function and biological processes. It is one of the most widely used systems for gene annotation [37]. Differentially expressed genes (DEGs) between the two risk subgroups were obtained using the ‘limma’, followed by GO analysis on the DEGs using the ‘clusterProfiler’, ‘org.Hs.eg.db’ and ‘DOSE’ packages. Enrichment results were mapped using the ‘ComplexHeatmap’, ‘ggpubr’, ‘circlize’, ‘RColorBrewer’ and ‘ggplot2’ packages to analyse the enrichment of DEGs between the risk subgroups in terms of cellular components (CC), molecular functions (MF) and biological processes (BP).

Furthermore, Gene Set Variation Analysis (GSVA) was mainly utilised to assess genomic enrichment in the transcriptome [38]. In this study, GSVA was used to obtain the Kyoto Gene and Genome Encyclopedia (KEGG) pathways enrichment in the two risk groups and further analyse the association between the KEGG pathways and lncRNA expressions in the PANRI. The process was implemented using the ‘limma’, ‘GSEABase’, ‘reshape2’, ‘GSVA’, ‘ggplot2’ and ‘pheatmap’.

### Association between the PANRI and TMB in HCC

The downloaded simple nucleotide variation data from the TCGA-LIHC cohort were collated using Perl scripts to obtain a TMB data matrix. The ‘limma’ and ‘ggpubr’ packages were utilised to analyse and visualise TMB differences between the risk subgroups. Moreover, K-M curves were utilised to explore survival differences between different TMB subgroups and different risk subgroup combinations. The 20 genes with the highest mutation frequency in the two risk subgroups were then extracted and a corresponding waterfall was plotted using the ‘maftools’ package.

### Predictive value of the PANRI in immune characteristics of HCC

To analyse the association between risk scores and immune characteristics, we performed a single sample gene set enrichment analysis (ssGSEA) using the ‘GSVA’ and ‘GSEABase’ packages, which determined the status of immune cell infiltration, calculated the proportion of different immune cells types in the tumour tissue in each sample and obtained the corresponding immune cell score and function score. Furthermore, the ‘pheatmap’, ‘ggpubr ‘and ‘reshape2’ packages were utilised to plot box plots of the differences in scores between different risk groups.

TIMER is a platform for the systematic investigation of immune infiltration in different types of cancer [39]. Using the TIMER2.0 platform, we acquired a matrix of immune cell infiltration data based on six algorithms for the TCGA-LIHC cohort. Additionally, the ‘ggplot2’, ‘ggpubr’, ‘ggtext’, ‘tidyverse’ and ‘scales’ were utilised to analyse and plot correlation bubble plots, thereby determining the correlation between risk scores and immune cells.

The ESTIMATE algorithm can estimate the number of normal and tumour cells in samples [40]. In the present study, the ‘ESTIMATE’ package was utilised to input the number of stromal cells and immune infiltrating cells in each sample in the TCGA-LIHC cohort and obtain the corresponding scores. The sum of the stromal score and the immune score was considered the ESTIMATE score. Finally, the ‘ggpubr’ was utilised to visualise the differences in scores between the two risk groups.

As a key regulatory factor of the immune system, the activation of immune checkpoints can induce tumour-related immune escape [41]. Therefore, we analysed the differences in different immune checkpoints between the two risk subgroups and visualised the outcomes using ‘ggplot2’ and ‘ggpubr’.

### The value of PANRI in predicting response to drug therapy for HCC

To investigate the potential value of the risk index in the clinical pharmacotherapy of HCC, the ‘pRRophetic’ package was utilised to calculate the IC50 of some therapeutic drugs in the different risk groups [42]. Furthermore, for drugs with significantly different IC50s in the two subgroups, box plots were plotted using the ‘ggpubr’.

### Consensus clustering analysis

The ‘ConsensusClusterPlus’ package was utilised to cluster patients with HCC based on the PANRI, and principal component analysis (PCA) was performed utilising the R packages ‘ggplot2’ and ‘Rtsne’. The prognostic and tumour immune microenvironment characteristics of patients with different HCC clusters were further analysed using K-M curves, ESTIMATE and immune checkpoints. Box plots of differential outcomes were plotted using the ‘ggplot2’, ‘ggpubr’ and ‘reshape2’ packages.

### Cell culture

Human HCC cell lines HepG2 were purchased from American Type Culture Collection (ATCC, USA). Cells were grown routinely in DMEM medium (Gibco, USA) containing 10% FBS (Gibco, USA) in a 37°C humidified 5% CO_2_ incubator.

### RNA extraction and RT-PCR

Total RNA was extracted from cells using DNAaway RNA Mini-Preps Kit (Sangon Biotech, China), and the NanoDrop 1000 was utilised to assess the quality of the extracted RNA. After reverse transcription, a PCR reaction system was prepared, and qPCR analysis was conducted by real-time detection system through SYBR green I dye (Applied Biosystems, USA) detection. The expression levels of AL049840.4 were quantified and calculated with the method of 2^−ΔΔCt^. The primer sequence is shown in Supplementary Table 2.

### Transfection of small interfering RNA (siRNA)

For the knockdown of AL049840.4, siRNA was transfected into HepG2 cells using Lipofectamine™ 3000 Transfection reagent (Thermo Fisher Scientific, USA). The final concentration of siRNA was 10 nM, and the volume of transfection reagent was used according to the supplier’s manual. The sequence of siRNA is shown in Supplementary Table 2.

### CCK-8 assay

The constructed siRNA-negative control (NC) cells and si-AL049840.4 cells were respectively seeded in 96-well culture plates (5 × 10^3^/well) and placed at 37°C, 5% CO_2_ incubator overnight. The CCK-8 Assay Reagent (Bimake, USA) was added to each well, and the assay was conducted according to the manufacturer’s instructions. A microplate reader (BioTek Instruments, USA) was used to measure the absorbance values at 450 nm, and to detect changes in cell proliferation ability of each group.

### Cell apoptosis assay

At 48 h after transfection, cells were harvested through trypsinization, and washed twice with PBS (KeyGen BioTECH, China, pH 7.2). The cells were centrifuged at 1000 g/min for 5 min, then the supernatant was discarded and the pellet was resuspended in 1 × binding buffer (Biolegend, USA) at a density of 1.0 × 10^5^ cells per ml. One hundred μL of the sample solution was transferred to a 5 mL culture tube, and incubated with 5 μl of FITC-conjugated annexin V (Biolegend) and 5 μl of PI (Biolegend) for 15 min. 400 μl of 1 × binding buffer was added to each sample tube, and fluorescence of stained cells was measured on FACSCanto II (BD Biosciences, USA) and analyzed with Flowjo.

### Transwell assay

The constructed NC, si-AL049840.4 cells were inoculated into the wells of Transwell plate containing serum-free DMEM medium (4 × 10^4^ cells/well). The lower well contains 800 μl of complete medium (DMEM and 10% FBS). After incubation at 37°C for 36 h, remove cells that have not migrated through the well with a cotton swab. Cells in the lower chamber were fixed with 4% methanal for 15 min and stained with 1% crystal violet in 2% ethanol for 10 min, photographed and counted.

### Statistical analysis

All statistical analyses were performed using R software (version 4.1.2) and the corresponding R packages. K-M method was utilised to plot the survival curves of different subgroups. The correlation between different continuous variables was assessed by Pearson correlation test. The Wilcoxon test was utilised for comparing two groups. *P* < 0.05 was considered as statistically significant for a difference.

### Data availability statement

The data sets used and/or analysed during the current study are available from the corresponding author.

## RESULTS

### PANoptosis-related lncRNAs in HCC

**Figure 1:**
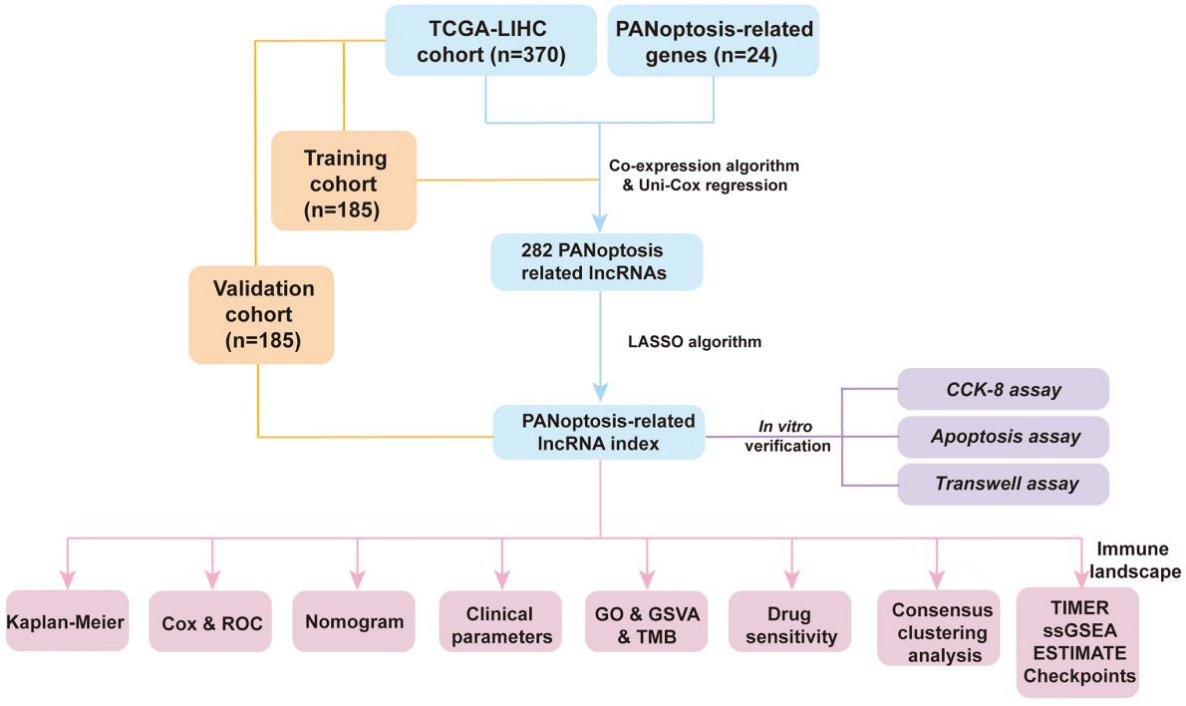
Flowchart of the present research.

**Figure 2:**
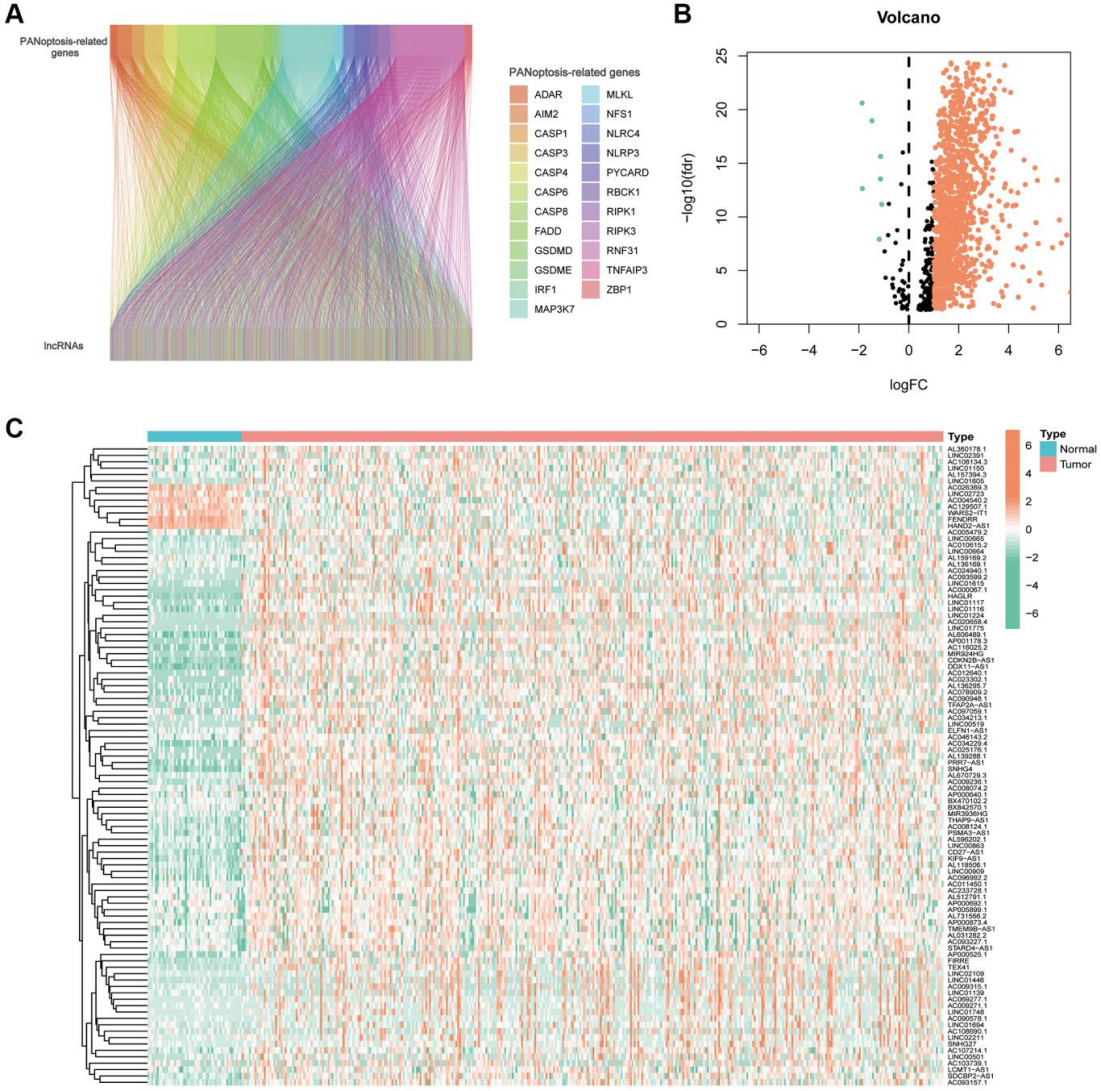
**PANoptosis-related lncRNAs in hepatocellular carcinoma.** (**A**) Sankey plots of the correlation between PANoptosis-related lncRNAs and PANoptosis-related genes. (**B**) Volcano plot showing 7 down-regulated and 1192 up-regulated expressed lncRNAs. (**C**) Heat map showing PANoptosis-related lncRNAs expressed in normal and tumour tissues.

Flowchart reflects the ideas and details of this research (Figure 1). Co-expression analysis identified 1476 PANoptosis-associated lncRNAs and their relationship with PRGs was presented using Sankey plots (Figure 2A). The volcano map showed that 1199 PANoptosis-related lncRNAs were differentially expressed in normal and tumour tissues (|Log2 FC| > 1, FDR < 0.05) (Figure 2B), of which seven lncRNAs were downregulated in tumours and the rest upregulated. Finally, the heat map revealed the expression status of the differentially expressed lncRNAs (Figure 2C).

### Establishment and validation of PANRI in HCC

**Figure 3:**
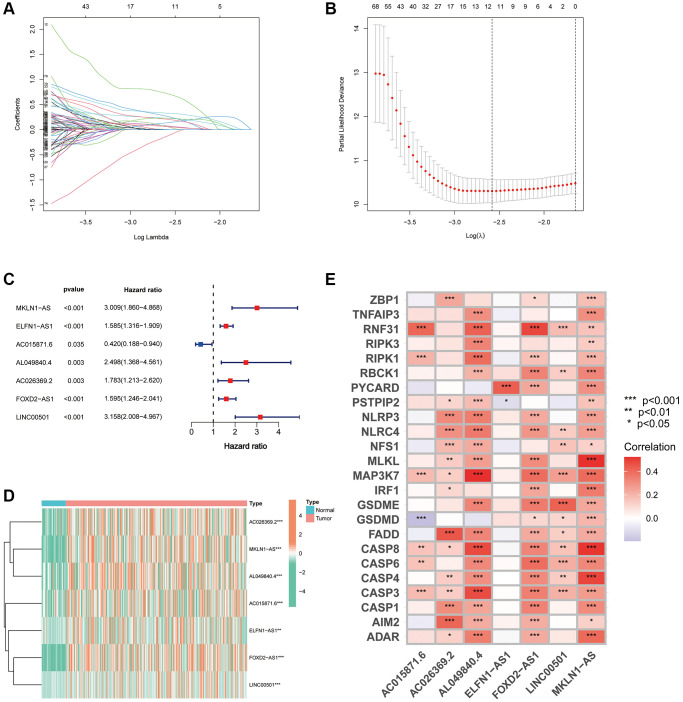
**Development of a PANRI in hepatocellular carcinoma.** (**A**, **B**) The LASSO coefficient and partial likelihood deviance of the PANRI. (**C**) A risk forest plot of the seven lncRNAs used to construct the PANRI. (**D**) Expression heat map of 7 lncRNAs used to construct PANRI. (**E**) Heat map of correlations between the expression of PANoptosis-related genes and the seven lncRNAs used to construct the PANRI.

All samples were further randomised into training and validation cohorts (Table 1). Using the uni-Cox regression algorithm, we obtained 282 PANoptosis-associated lncRNAs that were significantly associated with survival (*p* < 0.05), and the prognostic forest plots of these lncRNAs are presented in Supplementary Figure 1. Furthermore, using LASSO regression analysis (Figure 3A, 3B), seven lncRNAs were screened for use in constructing the risk index (Table 2). The risk score for each individual was calculated using the formula: Risk score = MKLN1-AS × (0.663709706) + ELFN1-AS1 × (0.335059408) + AC015871.6 × (−1.131739203) + AL049840.4 × (0.615830687) + AC026369.2 × (0.514830865) + FOXD2-AS1 × (0.288672846) + LINC00501 × (0.608378767). Figure 3C, 3D show the forest plot and expression heat map of the seven lncRNAs used to construct the index. Additionally, the expression correlation of the seven lncRNAs with 24 PRGs is also presented using a correlation heat map (Figure 3E).

**Table 1:** Clinicopathological parameters in the cohorts.

**Parameters**	**Type**	**Whole**	**Validation cohort**	**Training cohort**	***p*-value**
Age	≤65	232 (62.7%)	119 (64.32%)	113 (61.08%)	0.5909
	>65	138 (37.3%)	66 (35.68%)	72 (38.92%)	
Gender	Female	121 (32.7%)	62 (33.51%)	59 (31.89%)	0.8246
	Male	249 (67.3%)	123 (66.49%)	126 (68.11%)	
Grade	G1	55 (14.86%)	29 (15.68%)	26 (14.05%)	0.6058
	G2	177 (47.84%)	91 (49.19%)	86 (46.49%)	
	G3	121 (32.7%)	58 (31.35%)	63 (34.05%)	
	G4	12 (3.24%)	4 (2.16%)	8 (4.32%)	
	Unknown	5 (1.35%)	3 (1.62%)	2 (1.08%)	
Stage	Stage I	171 (46.22%)	82 (44.32%)	89 (48.11%)	0.3627
	Stage II	85 (22.97%)	50 (27.03%)	35 (18.92%)	
	Stage III	85 (22.97%)	41 (22.16%)	44 (23.78%)	
	Stage IV	5 (1.35%)	2 (1.08%)	3 (1.62%)	
	Unknown	24 (6.49%)	10 (5.41%)	14 (7.57%)	

**Table 2:** PANoptosis-related lncRNA index in HCC.

**lncRNA**	**Coef**	**HR**	***p*-value**
MKLN1-AS	0.663709706	3.009441601	<0.001
ELFN1-AS1	0.335059408	1.585022698	<0.001
AC015871.6	−1.131739203	0.419749336	0.035
AL049840.4	0.615830687	2.49788245	0.003
AC026369.2	0.514830865	1.78274389	0.003
FOXD2-AS1	0.288672846	1.594575947	<0.001
LINC00501	0.608378767	3.158318378	<0.001

**Figure 4:**
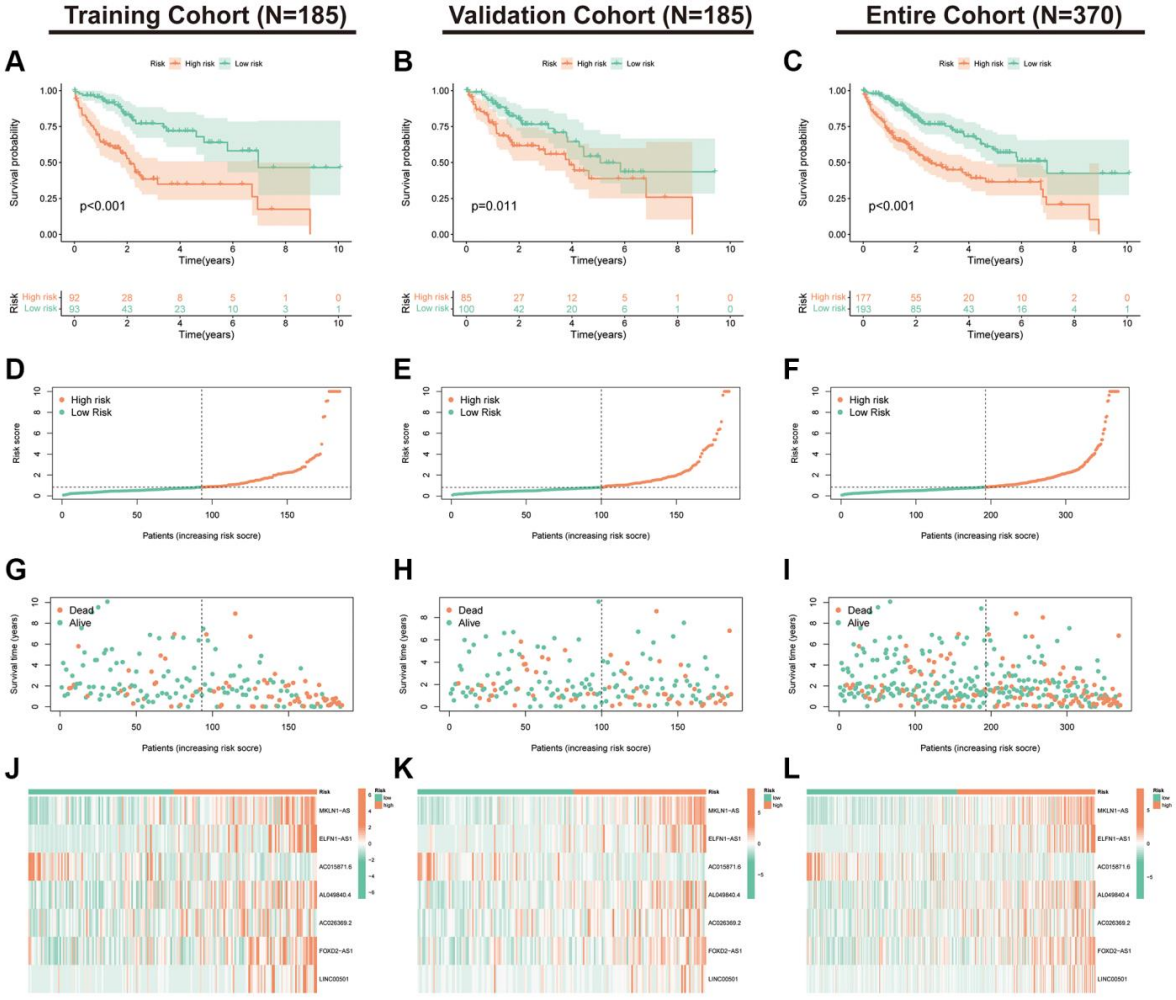
**Validation of the PANRI in hepatocellular carcinoma.** (**A**–**C**) Kaplan–Meier curves for overall survival in the training (*N* = 185), validation (*N* = 185) and entire (*N* = 370) cohorts. (**D**–**F**) Risk score distribution in the three cohorts. (**G**–**I**) Survival status in the three cohorts. (**J**–**L**) Heatmap of the expression of the seven PANoptosis-related lncRNAs in the three cohorts.

K-M curves indicated that the expression of seven index-related lncRNAs was correlated with the prognosis of HCC (Supplementary Figure 2). We further validated the stability of the index in the training, validation and TCGA-LIHC cohorts. First, we assessed the K-M curves, risk distribution and survival status of the patients in the three cohorts (Figure 4A–4I). The results revealed that the populations in the low-risk subgroup in all cohorts had a significantly better prognosis. Additionally, expression heat maps showed that AC015871.6 was lowly expressed in the high-risk subgroup in the training, validation and TCGA cohorts, while the remaining six lncRNAs were highly expressed in the high-risk subgroup (Figure 4J–4L).

### Evaluation of the PANRI in HCC

**Figure 5:**
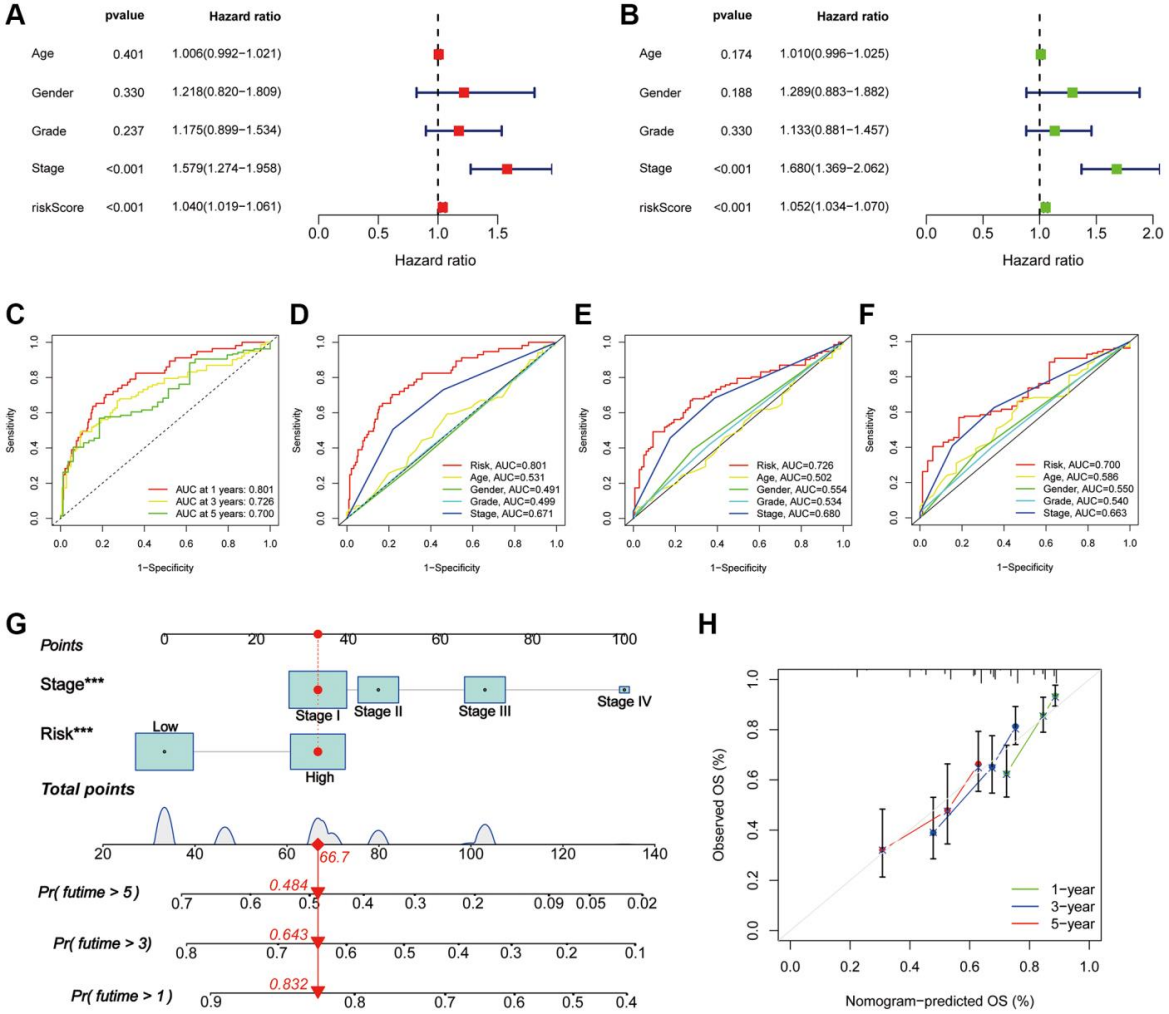
**Assessment of the PANRI in hepatocellular carcinoma.** (**A**, **B**) Risk forest plots for multivariate and univariate Cox regression. (**C**) ROC curves of the 1-, 3- and 5-year survival in the TCGA cohort. (**D**–**F**) Comparison of risk score ROC curves with clinicopathological parameter ROC curves. (**G**) Tumour stage and risk status were used to construct a nomogram for predicting patient survival. (**H**) Calibration curves for the nomogram. ^*^p < 0.05, ^**^p < 0.01 and ^***^p < 0.001.

To further assess the prognostic predictive value of PANRI, we first performed multi- and uni-Cox regression analyses. The outcomes indicated that the PANRI was an independent prognostic element with hazard ratios of 1.040 and 1.052 (Figure 5A, 5B). Additionally, the ROC curves revealed that the area under the curve (AUC) for PANRI at 1, 3 and 5 years was 0.801, 0.726 and 0.700 (Figure 5C). Moreover, the ROC curves also showed that PANRI had better predictive efficacy than clinicopathological parameters such as age, sex, tumour grade and tumour stage (Figure 5D–5F). Notably, differential expression analysis showed that 23 of the 24 PRGs were more highly expressed in the high-risk subgroup (Supplementary Figure 3), suggesting that the high-risk population may have a higher level of PANoptosis.

### Nomogram for patients with HCC

Based on multi-cox analyses, the tumour stage and risk status were incorporated into the construction of nomograms for the convenient clinical prediction of clinical outcomes for each patient with HCC (Figure 5G). The corresponding scores for each indicator are presented in the nomogram and all scores are summed as a tool to predict patient prognosis. The nomogram estimated the 1-, 3-, and 5-year OS rates for an individual (high-risk, stage I) to be 0.832, 0.643, and 0.484, respectively. Furthermore, the calibration curves of survival indicated excellent agreement between the actual and nomogram-predicted outcomes (Figure 5H).

### Association of PANRI with clinicopathological parameters in HCC

**Figure 6:**
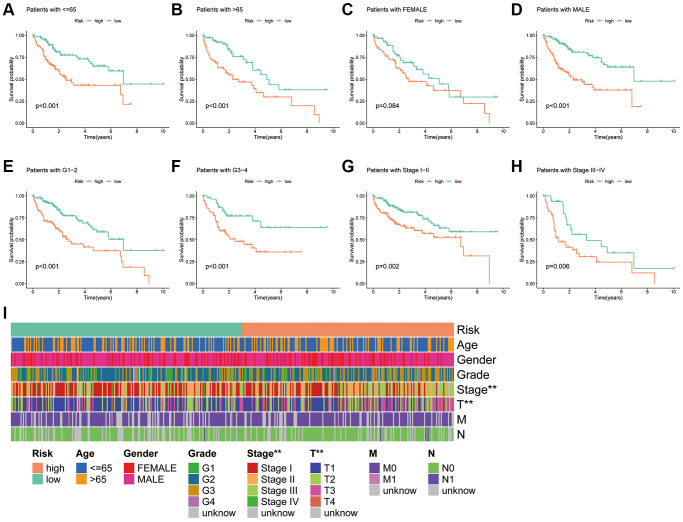
**Association of the PANRI with clinicopathological features in hepatocellular carcinoma.** (**A**–**H**) Kaplan–Meier curves stratified by age, gender, tumour grade and tumour stage. (**I**) Heat map of the distribution of clinical parameters in different risk groups. ^*^p < 0.05, ^**^p < 0.01 and ^***^p < 0.001.

As shown in the K-M survival curves (Figure 6A–6H), individuals with HCC of different ages, grades and stages had better survival rates in the low-risk group. Although the K-M curves for women did not differ significantly in the two risk groups, a trend towards a separation of the curves could be seen. The findings demonstrate the broad applicability of PANRI in patients with HCC having different clinical features. Additionally, the heat map of the clinical parameter status of patients in different risk groups revealed that tumour stage and T-stage differed between the risk subgroups (Figure 6I).

### PANRI-related mechanisms

**Figure 7:**
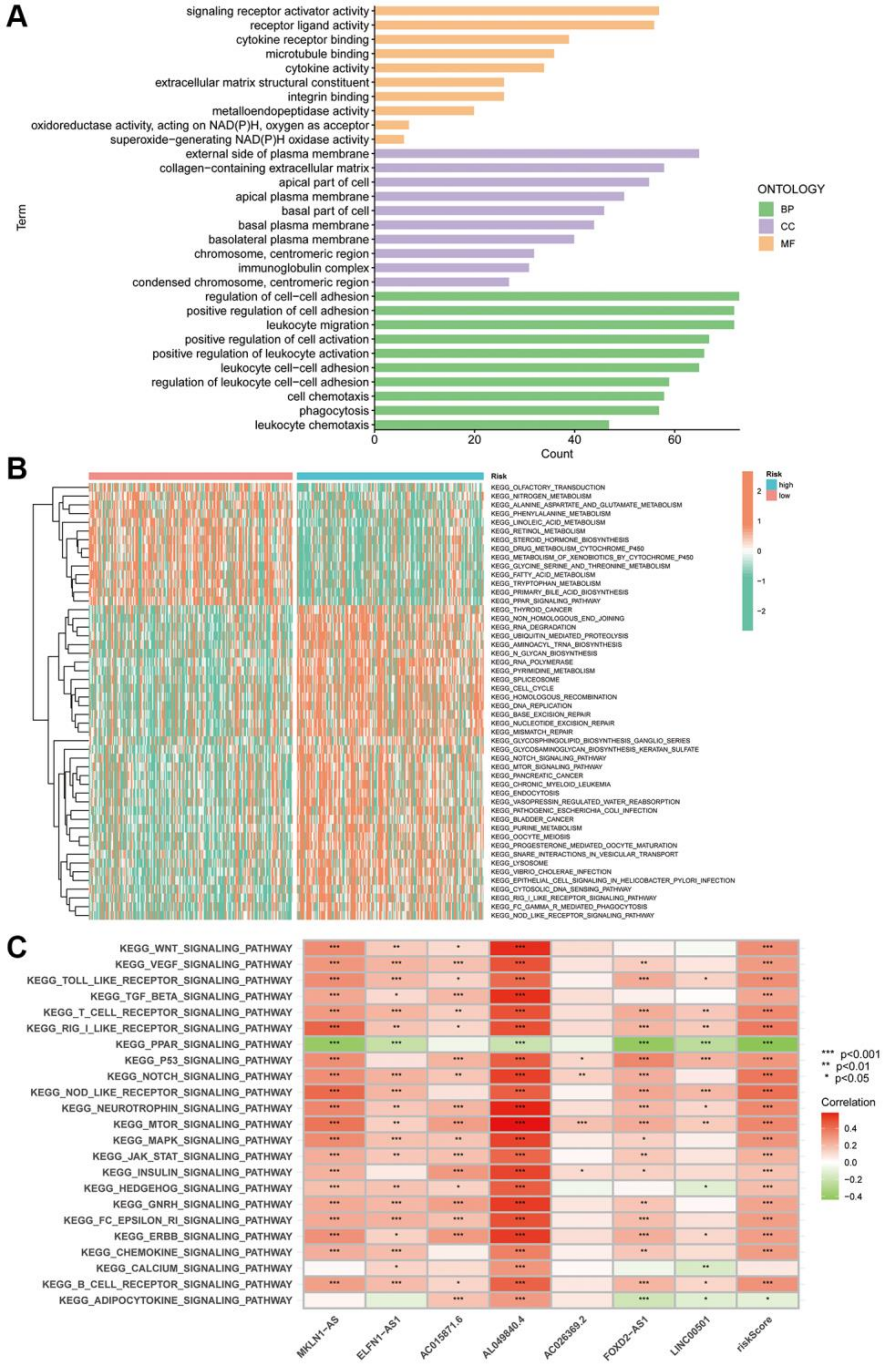
**PANRI-based GO and KEGG analysis.** (**A**) GO analysis shows the enrichment of DEGs between the risk groups. (**B**) Heat map of functional pathway enrichment differences between the risk groups. (**C**) Heat map of the spearman correlation analysis between the expression of the seven lncRNAs involved in the model construction and tumour-related pathways.

Given the differences in the prognosis of individuals in the different risk subgroups, we performed GO, GSVA and KEGG enrichment exploration to further investigate the possible mechanisms. GO enrichment analysis revealed that in terms of biological process, DEGs in the two risk groups were mainly enriched in the regulation of cell-cell adhesion, positive regulation of cell adhesion and migration of leukocytes. In terms of cellular composition, DEGs were mainly enriched in the external side of the plasma membrane, collagen-containing extracellular matrix and apical part of the cell. In terms of molecular function, DEGs were mainly enriched in the ECM structure, mainly in signalling receptor activator activity and cytokine receptor binding (Figure 7A).

GSVA explored the differences in KEGG pathways between the risk subgroups. The heat map revealed that the functions enriched in the high-risk subgroup included RNA degradation, ubiquitin-mediated protein hydrolysis, RNA polymerase, pyrimidine metabolism, homologous recombination, DNA replication, mismatch repair, nucleotide excision repair and Notch and mTOR pathways. Notably, these functions are widely involved in the evolution of tumour biological behaviour. Additionally, nitrogen metabolism, phenylalanine metabolism, fatty acid generation, primary bile acid biosynthesis, steroid hormone biosynthesis and linoleic acid metabolism were enriched in the low-risk subgroup (Figure 7B). Finally, spearman correlation analysis heat map showed a broad correlation between the expression of these seven PANoptosis-related lncRNAs and signalling pathways (Figure 7C).

### Association of the PANRI with TMB in HCC

**Figure 8:**
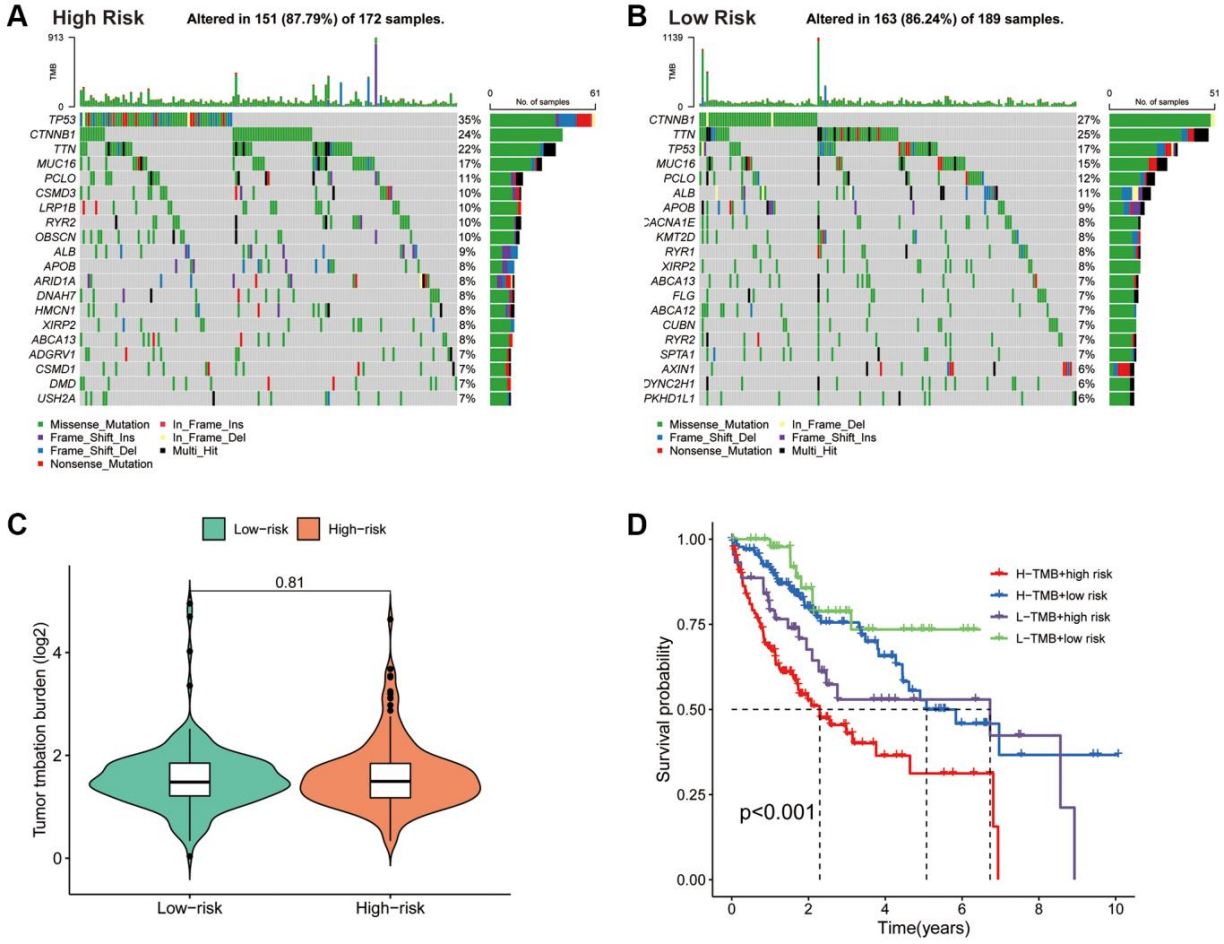
**Association of the PANRI with TMB in hepatocellular carcinoma.** (**A**) Mutation waterfall map showing the 20 genes with the highest mutation frequency in the high-risk group. (**B**) Mutation waterfall map showing the 20 genes with the highest mutation frequency in the low-risk group. (**C**) Comparison of TMB between the two risk groups. (**D**) Kaplan–Meier curves for the TMB subgroups combined with the risk subgroups.

The mutation waterfall plot shows the top 20 genes with the highest mutation frequencies in the two risk subgroups (Figure 8A, 8B). In the high-risk population, the top five genes with the highest mutation frequencies were *TP53* (35%), *CTNNB1* (24%), *TTN* (22%), *MUC16* (17%) and *PCLO* (11%), while in the low-risk population included *CTNNB1* (27%), *TTN* (25%), *TP53* (17%), *MUC16* (15%) and *PCLO* (12%).

Tumours with high levels of TMB tend to have higher neoantigen levels, which stimulate the proliferation of anti-tumour effector immune cells and serve as a predictive biomarker for the efficacy of immunotherapeutic response to ICIs in some tumours [43]. Although there was no significant difference in TMB levels between the two risk subgroups (Figure 8C), significant differences in survival between the different four subgroups were observed (Figure 8D). These findings suggest that the combination of the PANRI and TMB levels can better predict the prognosis in HCC.

### Role of PANRI in predicting immune characteristics in HCC

**Figure 9:**
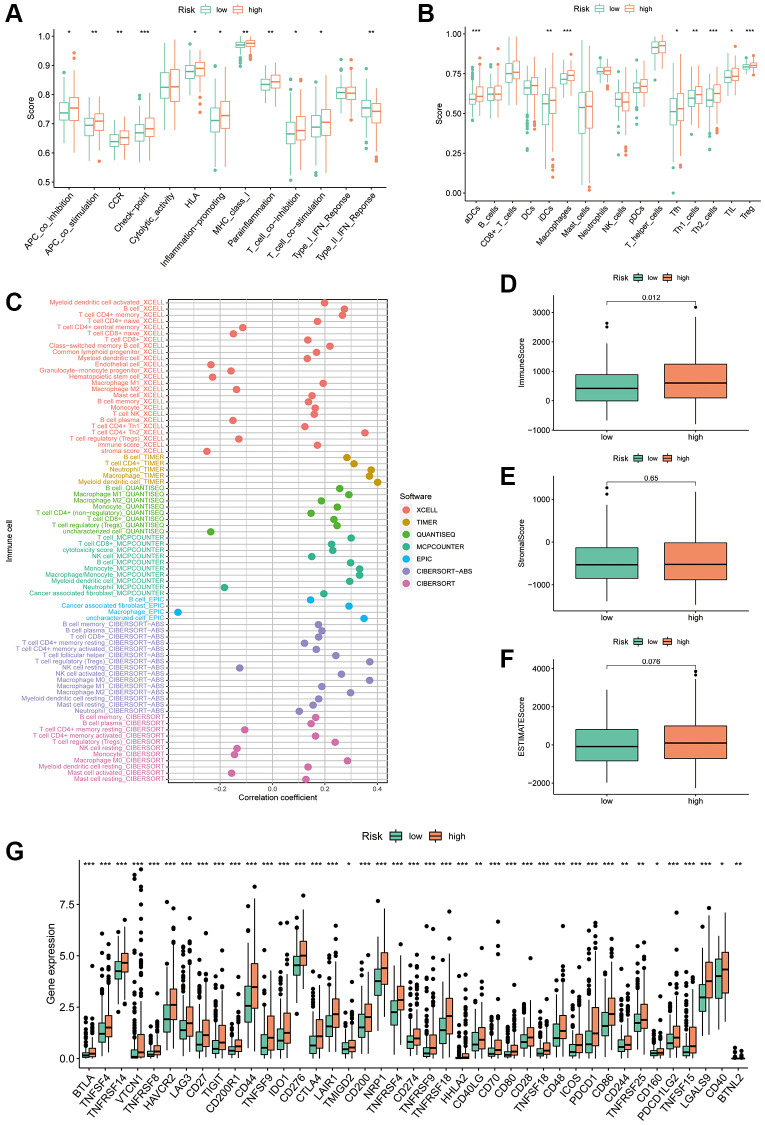
**Correlation of the PANRI with TIME in hepatocellular carcinoma.** (**A**) Box plots of differences in immune-related functions between the high- and low-risk subgroups. (**B**) Box plots of differences in immune cell scores between the high- and low-risk subgroups. (**C**) Bubble plots illustrate the correlation between immune cells and risk scores. (**D**–**F**) Box plots of the differences in the immune cell, stromal cell scores and ESTIMATE scores in the different risk cohorts. (**G**) Heat map of the differences in immune checkpoints between the two risk subgroups. ^*^p < 0.05, ^**^p < 0.01 and ^***^p < 0.001.

The TIME has been closely linked to tumour evolution and influences the response of patients with tumours to immunotherapy. Moreover, there is growing evidence that PCD plays an important role in regulating TIME [33]. To further explore the predictive potential of PANRI in TIME, we utilised the ssGSEA algorithm. Box plots indicated that most immune-related functions including antigen-presenting cell co-inhibition and co-stimulation, immune checkpoint, human leukocyte antigen, inflammation promotion, T-cell co-stimulation and T-cell co-inhibition were stronger in the high-risk population (Figure 9A). In terms of immune cells, immature dendritic cells, macrophages, activated dendritic cells, follicular helper T cells, helper T cells, tumour infiltrating lymphocytes and regulatory T cells were highly expressed in the high-risk subgroup (Figure 9B). Bubble plots showed a positive correlation between most immune infiltrating cells and risk scores (Figure 9C). Furthermore, ESTIMATE analysis revealed that while stromal and ESTIMATE scores did not differ significantly between the two risk groups, immune scores were significantly higher in the high-risk population (Figure 9D–9F).

The key targets in the clinical use of ICIs are currently PD-1, PD-L1 and CTLA-4 [44]. To verify the predictive value of PANRI in the treatment response of ICIs, we analysed the association between immune checkpoints and PANRI. The results showed that most immune checkpoints, including PD-1, PD-L1 and CTLA-4, were more highly expressed in the high-risk group (Figure 9G).

### Relationship between the PANRI and HCC therapy

**Figure 10:**
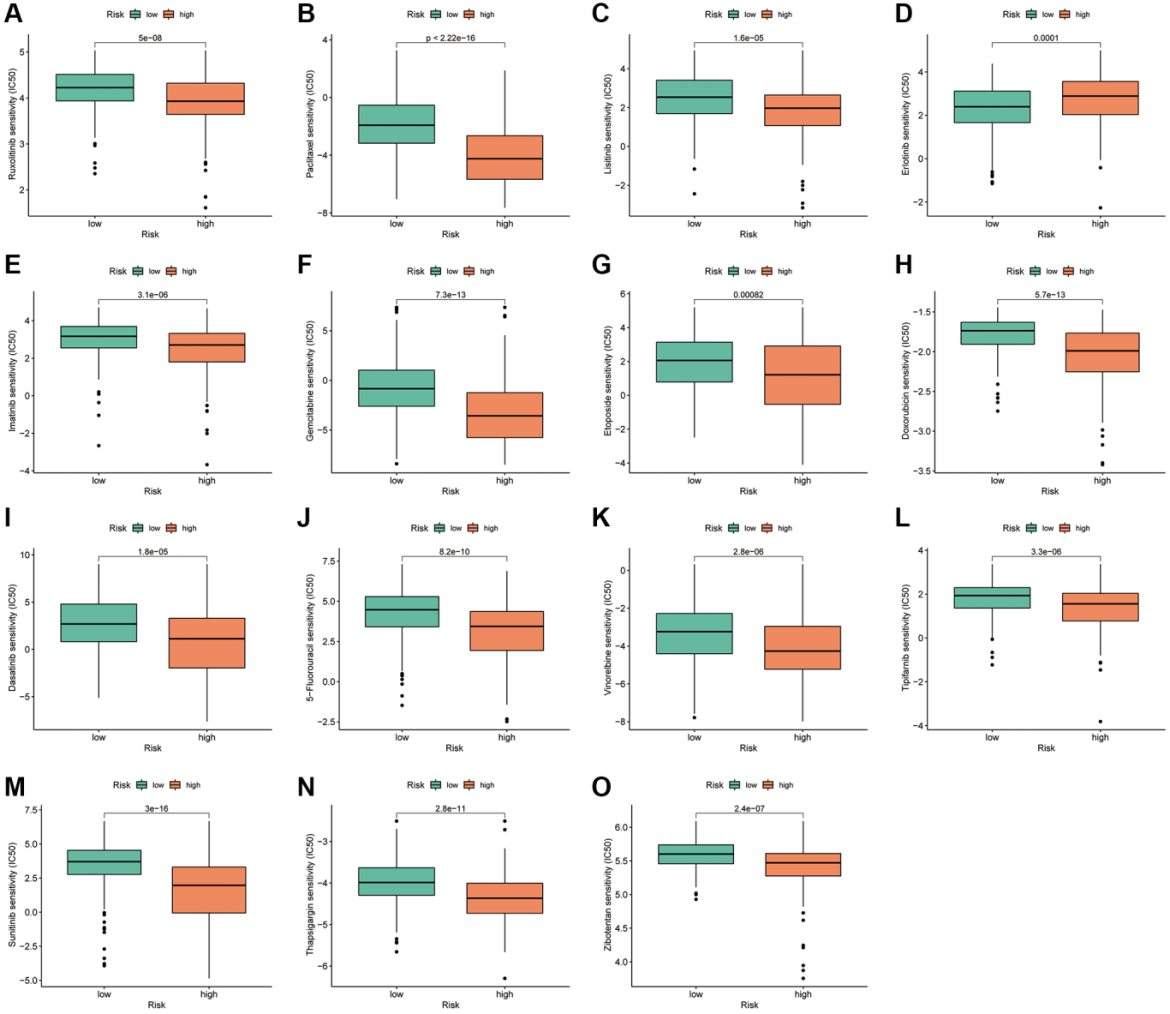
**PANRI-based drug sensitivity analysis.** (**A**–**O**) Box plots show that the IC50 for certain clinical therapeutics differed significantly between the two risk subgroups (*p* < 0.001).

To analyse the therapeutic significance of PANRI in clinical drug selection, a sensitivity analysis of different drugs was performed using the ‘pRRophetic’ algorithm. The IC50 values of specific clinical therapeutics differed between the two risk subgroups (*p* < 0.001) (Figure 10A–10O). Among them, the targeted drugs, namely ruxolitinib, lisitinib, imatinib, dasatinib, tipifarnib, sunitinib and thapsigargin, and the chemotherapeutic drugs, namely gemcitabine, paclitaxel, etoposide, doxorubicin, 5-fluorouracil and vinorelbine, had lower IC50s in the high-risk subgroup. However, contrasting results were observed for erlotinib, a drug targeting the epidermal growth factor receptor (EGFR).

### Cluster typing based on the PANRI

**Figure 11:**
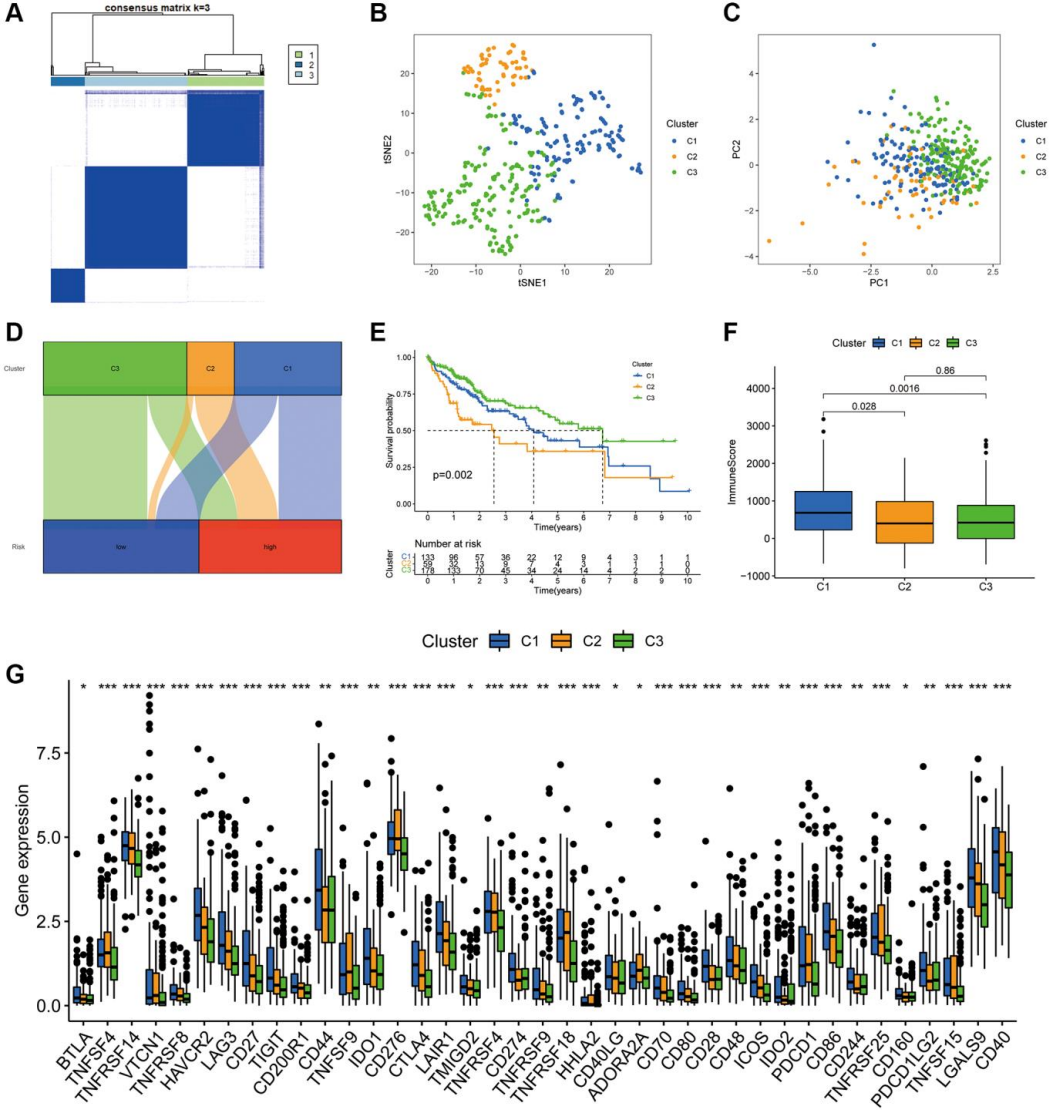
**Hepatocellular carcinoma classification based on the PANRI.** (**A**) Patients were divided into three clusters based on the consensus clustering matrix. (**B**–**C**) tSNE and PCA analyses of the three clusters. (**D**) Sankey plot for the risk cohorts and three clusters. (**E**) Kaplan–Meier curves of the three clusters. (**F**) Box plots of the differences in immune cell scores in the three clusters. (**G**) Expression of immune checkpoints in the three clusters. ^*^p < 0.05, ^**^p < 0.01 and ^***^p < 0.001.

According to the delta area plots and tracing plots of the consensus clustering algorithm, the lowest intra-cluster difference is found when k = 3 (Supplementary Figure 4). Herein, consensus clustering analysis based on the predictive index was used to classify patients into three clusters (Figure 11A). The tSNE and PCA analysis distinguished the distributional profiles of the three clusters (Figure 11B, 11C). Sankey plots showed that the majority of patients in clusters 1 and 2 were in the high-risk subgroup, while those in cluster 3 were in the low-risk subgroup (Figure 11D). K-M curves indicated that patients in cluster 3 had the best prognosis, followed by cluster 1; however, those in cluster 2 had the worst prognosis (*p* = 0.002) (Figure 11E). Furthermore, ESTIMATE analysis revealed that samples in cluster 1 had higher immune scores than clusters 2 and 3 (Figure 11F). In terms of immune checkpoints, most immune checkpoints were significantly more highly expressed in cluster 1 (Figure 11G), which, combined with the immune score results, suggests that cluster 1 could be a superior population for ICIs treatment. Thus, the consensus clustering analysis based on PANRI may not only determine prognosis but also aid in TIME characteristic identification.

### Experimental validation *in vitro*

**Figure 12:**
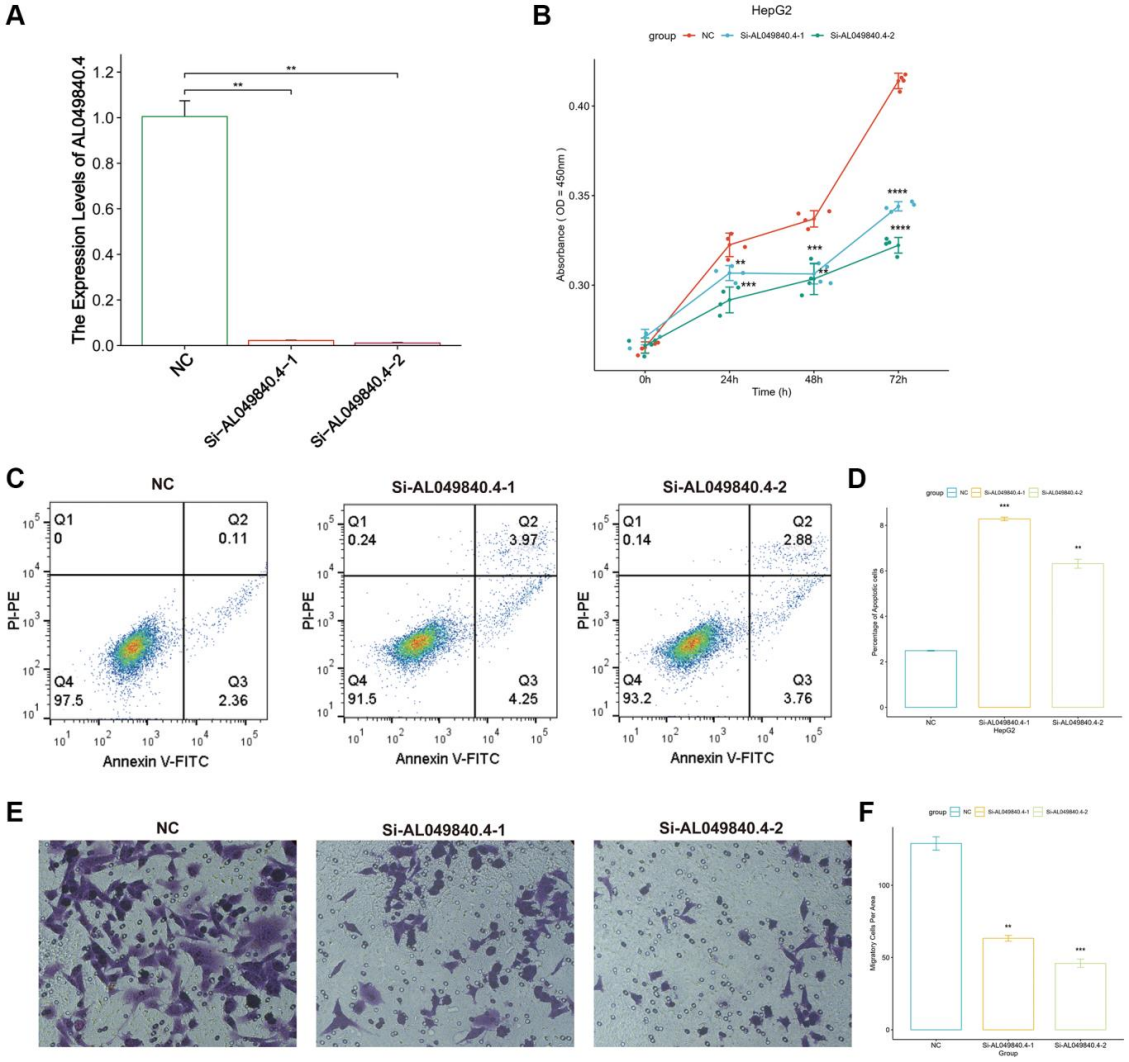
**Unfavorable impact of AL049840.4 on HCC *in vitro*.** (**A**) Validation of knockdown efficiency of AL049840.4 expression in HepG2 cells by qRT-PCR. (**B**) Cell viability of HepG2 cells after silencing AL049840.4 was detected by CCK-8 assay. (**C**, **D**) Cell apoptosis assay revealed that knockdown of AL049840.4 promoted apoptosis in HepG2 cells. (**E**, **F**) Transwell assay showed a statistically significant decrease in the HepG2 cells migration after AL049840.4 knockdown.

In PANRI, AL049840.4 belongs to the risk genes and is significantly associated with the expression of 20 PANoptosis-related genes. Additionally, GSVA results showed that the expression of AL049840.4 in HCC was significantly associated with several tumor-related signaling pathways, however, its role in HCC remains unreported. To know the effect of AL049840.4 on HCC, we further validated it in *in vitro* experiments. RT-PCR results showed that the transfection of siRNA successfully interfered with the mRNA expression of AL049840.4 in HepG2 cells (Figure 12A). Results of CCK-8 assay showed that knockdown of AL049840.4 suppressed the viability of HepG2 cells (Figure 12B). Additionally, results of cell apoptosis assay revealed that knockdown of AL049840.4 promoted apoptosis in HepG2 cells (Figure 12C, 12D). Furthermore, transwell assay showed a statistically significant decrease in the HepG2 cells migration after AL049840.4 knockdown (Figure 12E, 12F). Together, these results suggest that the expression of AL049840.4 is associated with the viability, migration and apoptosis of HCC cells.

## DISCUSSION

Although apoptosis, necroptosis and pyroptosis have been identified as independent forms of PCD, there is accumulating evidence of significant interconnections between these PCD types. PANoptosis, a unique type of PCD, is controlled by the PANoptosome and combines pyroptosis, apoptosis and necroptosis. However, PANoptosis cannot be defined solely based on a single type of cell death [45]. Currently, intensive research on PANoptosis is focused on the field of infection [46, 47], and PANoptosis-related tumour research is still in its infancy. Recent studies report that inflammatory cell death in PANoptosis could elicit an immune response [48], which is critical for tumour immunity and growth [12, 49]. Furthermore, the inhibition of PANoptosis promotes tumour progression [50]. Therefore, the study of the interaction between PANoptosis and TIME has the potential as a research hotspot in the field of oncology, considering the close interaction between tumour immunity and infection immunity [13, 51].

lncRNAs typically act as competitive endogenous RNAs regulating mRNA translation. They are also involved in the complex regulatory network of cancer development and regulate different types of PCD [52–54]. Furthermore, there is growing evidence supporting the function of lncRNAs as key regulators of HCC [55, 56], and potential diagnostic and prognostic markers of HCC [57]. Therefore, further elucidation of the role of lncRNAs in HCC could contribute to the current understanding of HCC progression and also to the determination of patient prognosis and selection of clinical treatment strategies.

In the present study, we established a PANRI to predict the clinical outcomes and immune microenvironment landscape of individuals with HCC based on PANoptosis-related lncRNAs. The results indicated that PANRI is an independent prognostic indicator of HCC, with better predictive efficacy than other clinicopathological parameters. Among the seven lncRNAs involved in the construction of PANRI, MKLN1-AS was demonstrated to promote tumour evolution and epithelial cell mesenchymal transition in HCC. It is also considered a potential target for HCC therapy [58, 59]. ELFN1-AS1 is speculated to be associated with cellular autophagy in HCC and affects patient prognosis [60]. Additionally, FOXD2-AS1 exhibits aberrant expression in various malignancies and promotes HCC invasion and migration by sponging miR-206 and regulating MAP3K1 expression [61, 62]. Moreover, LINC00501 has been demonstrated to correlate strongly with the clinical features of patients with HCC and is considered a potential biomarker for HCC [63]. Additionally, although AL049840.4 was shown to be a pyroptosis-associated lncRNA in HCC [64], its role in HCC has not been reported. In this study, the expression of AL049840.4 was shown to be significantly associated with 20 PANoptosis-related genes and multiple tumor-associated signaling pathways including WNT, P53, Notch, mTOR, MAPK and JAK-STAT. Additionally, our study revealed for the first time that AL049840.4 was associated with apoptosis, migration and viability of HepG2 cells. The deep mechanism of AL049840.4 in regulating PANoptosis in HCC deserves further exploration in the future.

The use of ICIs has ushered in a new era of tumour immunotherapy. Unlike conventional tumour treatments, such as surgery, radiotherapy and chemotherapy, ICIs can de-activate the immune checkpoints, restore the recognition of tumour cells by the immune system and enhance the killing of tumour cells by effector immune cells. However, the theoretical mechanisms are yet to be demonstrated in the clinical efficacy of ICIs, and the overall efficacy of different ICIs in tumours remains low. This low efficacy has been attributed to the low infiltration of effector T cells in the tumour tissue, and these tumours are classified as ‘cold immune tumours’ [65, 66]. Conversely, ‘hot immune tumours’ are characterised by an immune microenvironment consisting of activated immune checkpoints and a high infiltration of immune cells, thereby responding better to ICIs [65, 67]. Therefore, the determination of tumour immunophenotypes is important for determining and predicting the efficacy of ICIs in patients with cancer. Notably, most of the immune checkpoints were highly expressed in the high-risk subgroup, signifying a highly immunosuppressed state in this group, which could partially explain the poor prognosis of the high-risk subgroup. ssGSEA and ESTIMATE results suggest that the high-risk group not only has a highly activated state of immune checkpoints but also a high state of immune cell infiltration. Together, these findings suggest that the patients in the high-risk population are more likely to benefit from treatment with ICIs than the low-risk patients. TMB is another clinically used biomarker that has been used to predict the efficacy of ICIs for certain cancer types [68–70]. However, the TMB threshold that is currently valid for predicting the efficacy of ICIs requires further validation in a large number of prospective clinical studies [68]. In the present study, PANRI combined with TMB levels better predicted the prognosis of individuals with HCC although TMB did not differ significantly between risk groups.

The PANRI provides a basis for the personalised selection of targeted and chemotherapeutic agents, thereby guiding the personalised treatment of patients with HCC. Among these, a meta-analysis revealed that erlotinib, a TKI targeting EGFR, in combination with the anti-angiogenesis-targeting drug bevacizumab was effective in the 2nd-line treatment of HCC [71]. IC50 values indicate that the low-risk groups may benefit more from erlotinib. Additionally, dasatinib in combination with sorafenib has been reported to exert a synergistic inhibitory effect on tumour migration and angiogenesis in HCC [72]. The results suggest that the high-risk groups are more likely to benefit from dasatinib. In terms of chemotherapeutic drugs, the IC50 of chemotherapeutic agents including paclitaxel, gemcitabine, etoposide, doxorubicin, 5-Fluorouracil and vinorelbine in the low-risk group was higher than that in the high-risk group, suggesting that the high-risk population could benefit more from chemotherapy.

Molecular subtypes of tumours contribute to the differentiation of patients' prognosis and TIME characteristics [73–75]. To explore the differences in survival and TIME features of patients with different subtypes of HCC, we divided the samples into three clusters according to consensus clustering algorithm. There were significant differences in survival among patients in the three clusters, with cluster 3 having the best survival and cluster 2 having the worst prognosis. In terms of TIME, the immune infiltration status of cluster 1 was higher than that of the other two clusters. Moreover, most of the immune checkpoints also presented a higher level of expression in cluster 1. Overall, our cluster analysis also aids in predicting the prognosis and TIME landscape in HCC.

Although the PANRI developed in the present study has been validated and evaluated by different methods, there are still some limitations. First, the clinical application of lncRNA as a biomarker has not yet been popularized, and the clinical predictive efficacy of PANRI still needs to be further confirmed in prospective clinical studies with large sample sizes in the future. Additionally, the regulatory mechanism of PANRI-associated lncRNAs in PANoptosis still needs to be further clarified in subsequent studies.

## CONCLUSION

To the best of our knowledge, this is the first risk evaluation index in HCC that is constructed based on PANoptosis-related lncRNAs. The PANRI and clusters identified herein can effectively predict the prognosis and TIME in HCC and offer a basis for the selection of individualized treatment regimens in the clinical setting.

## Supplementary Materials

Supplementary Figures

Supplementary Tables
