# Supplementary material for: A novel PANoptosis-related long non-coding RNA index to predict prognosis, immune microenvironment and personalised treatment in hepatocellular carcinoma
Source: Aging (Albany NY). 2024 Jan 26;16(3):2410–37. doi: 10.18632/aging.205488 (PMC10911344; doi:10.18632/aging.205488)
Supplement: Supplementary Figures [file aging-16-205488-s001.pdf]

SUPPLEMENTARY FIGURES

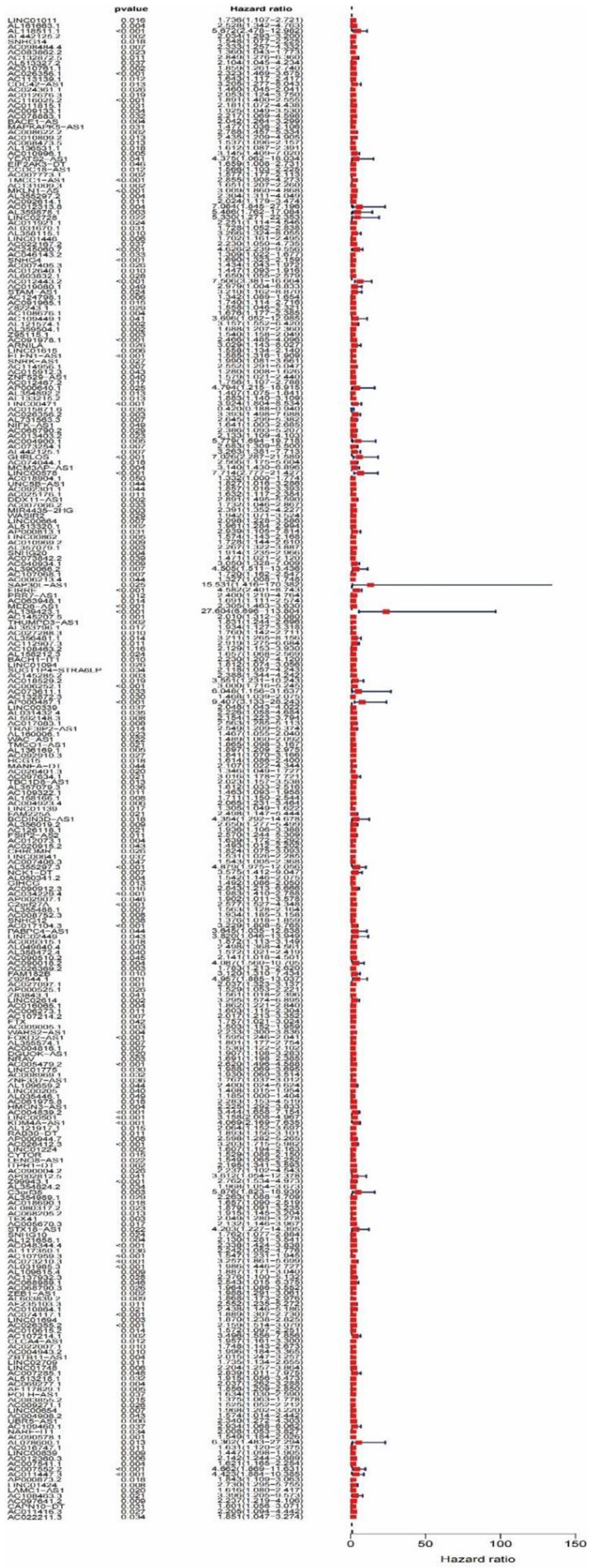

Supplementary Figure 1. 282 PANoptosis-associated lncRNAs significantly associated with survival.

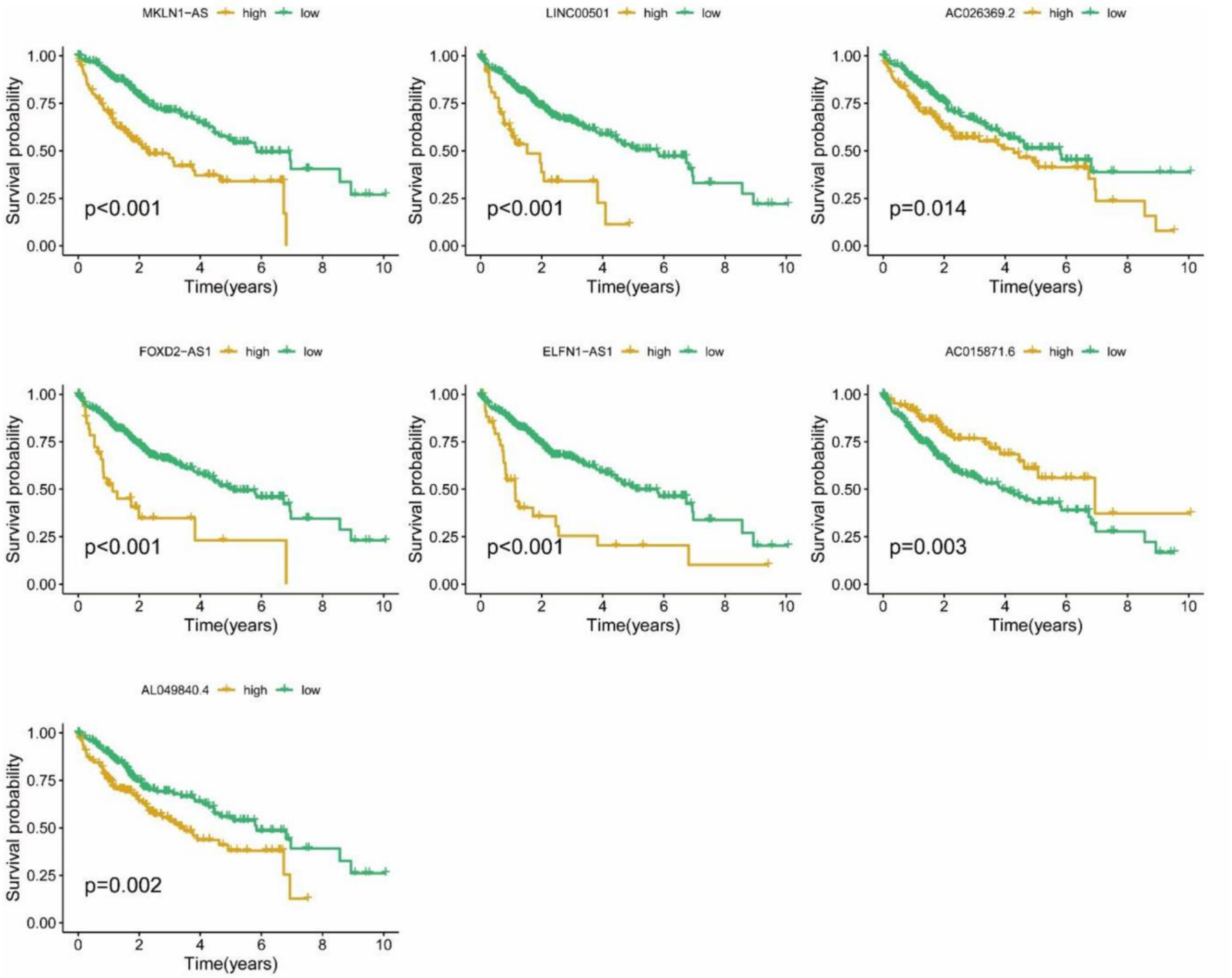

Supplementary Figure 2. K-M curves for the seven PANRI-associated lncRNAs.

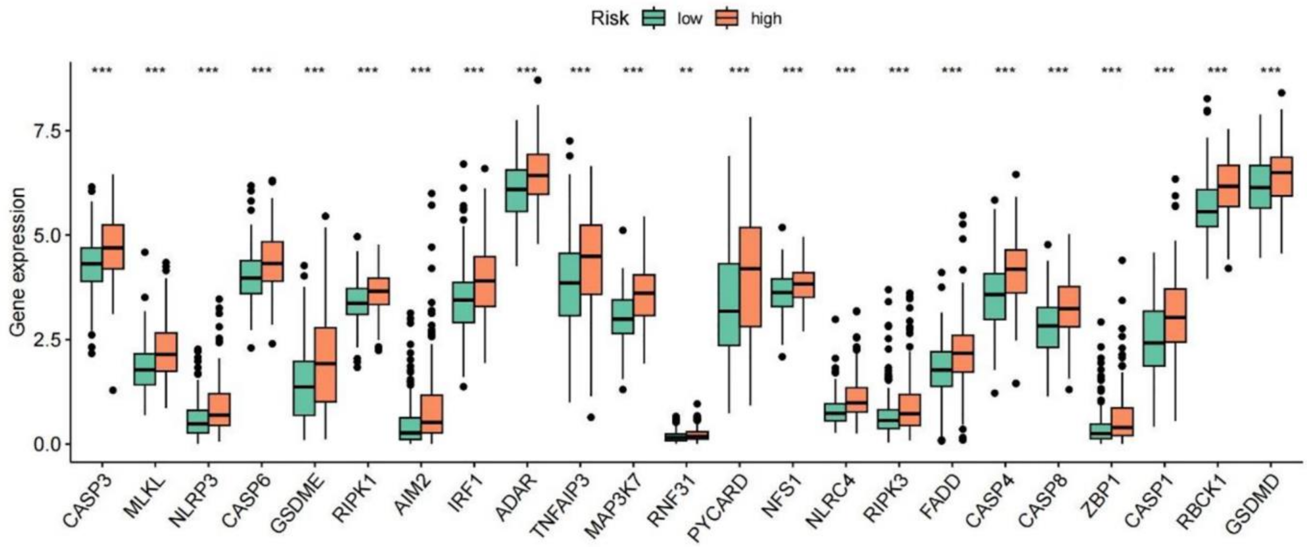

Supplementary Figure 3. Correlation of high- and low-risk subgroups with the expression levels of PANoptosis genes.

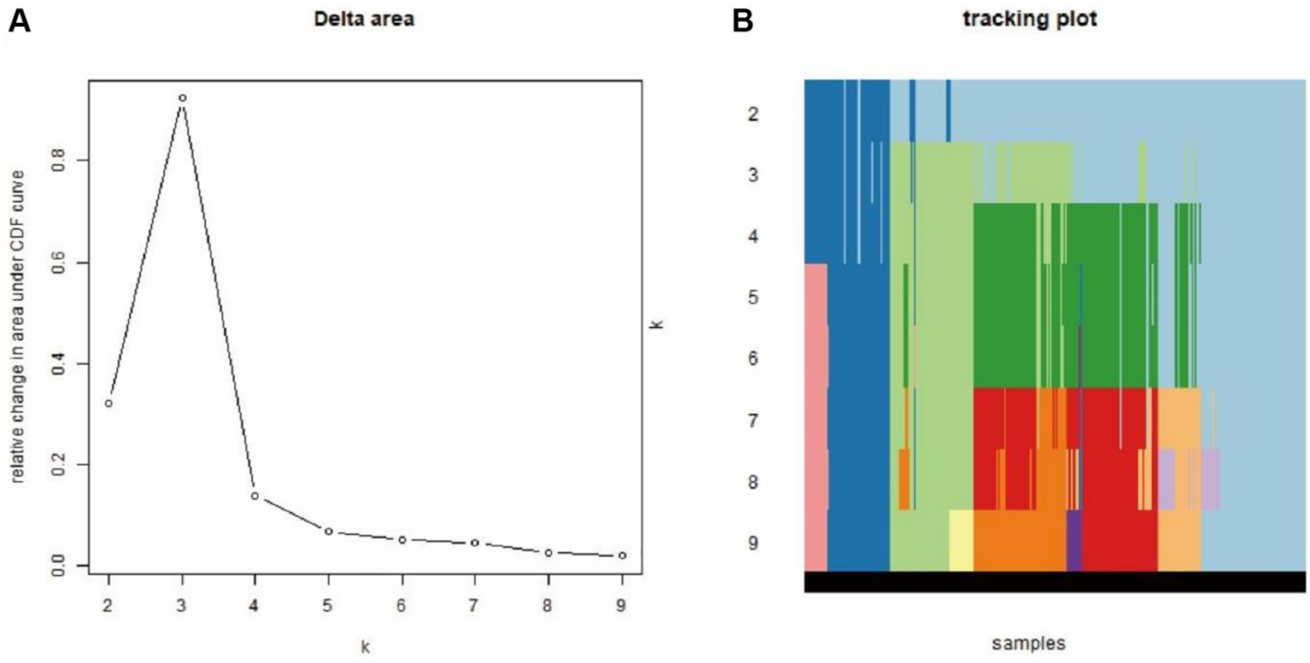

Supplementary Figure 4. Consensus clustering analysis (CCA). (**A**) Delta area plots (k = 2–9). (**B**) Tracling plots for CCA (k = 2–9).
