# Supplementary material for: A novel PANoptosis-related long non-coding RNA index to predict prognosis, immune microenvironment and personalised treatment in hepatocellular carcinoma
Source: Aging (Albany NY). 2024 Jan 26;16(3):2410–37. doi: 10.18632/aging.205488 (PMC10911344; doi:10.18632/aging.205488)
Supplement: Supplementary Tables [file aging-16-205488-s002.pdf]

SUPPLEMENTARY TABLES

Supplementary Table 1. PANoptosis-related genes.

PRGs	Source
ADAR	Karki, R., Sundaram, B., Sharma, B.R., et al. (2021). ADAR1 restricts ZBP1-mediated immune response and PANoptosis to promote tumorigenesis. Cell Rep 37(3), 109858. https://doi.org/10.1016/j.celrep.2021.109858.
AIM2	Lee, S., Karki, R., Wang, Y., et al. (2021). AIM2 forms a complex with pyrin and ZBP1 to drive PANoptosis and host defence. Nature 597(7876), 415-419. https://doi.org/10.1038/s41586-021-03875-8.
CASP1	Zheng, M., Karki, R., Vogel, P., et al. (2020). Caspase-6 Is a Key Regulator of Innate Immunity, Inflammasome Activation, and Host Defense. Cell 181(3), 674-687.e613. https://doi.org/10.1016/j.cell.2020.03.040.
CASP4	Christgen, S., Zheng, M., Kesavardhana, S., et al. (2020). Identification of the PANoptosome: A Molecular Platform Triggering Pyroptosis, Apoptosis, and Necroptosis (PANoptosis). Front Cell Infect Microbiol 10, 237. https://doi.org/10.3389/fcimb.2020.00237.
CASP3	Zheng, M., Karki, R., Vogel, P., et al. (2020). Caspase-6 Is a Key Regulator of Innate Immunity, Inflammasome Activation, and Host Defense. Cell 181(3), 674-687.e613. https://doi.org/10.1016/j.cell.2020.03.040.
CASP6	Zheng, M., Karki, R., Vogel, P., et al. (2020). Caspase-6 Is a Key Regulator of Innate Immunity, Inflammasome Activation, and Host Defense. Cell 181(3), 674-687.e613. https://doi.org/10.1016/j.cell.2020.03.040.
CASP8	Malireddi, R.K.S., Kesavardhana, S., and Kanneganti, T.D. (2019). ZBP1 and TAK1: Master Regulators of NLRP3 Inflammasome/Pyroptosis, Apoptosis, and Necroptosis (PAN-optosis). Front Cell Infect Microbiol 9, 406. https://doi.org/10.3389/fcimb.2019.00406.
FADD	Malireddi, R.K.S., Kesavardhana, S., and Kanneganti, T.D. (2019). ZBP1 and TAK1: Master Regulators of NLRP3 Inflammasome/Pyroptosis, Apoptosis, and Necroptosis (PAN-optosis). Front Cell Infect Microbiol 9, 406. https://doi.org/10.3389/fcimb.2019.00406.
GSDMD	Zheng, M., Karki, R., Vogel, P., et al. (2020). Caspase-6 Is a Key Regulator of Innate Immunity, Inflammasome Activation, and Host Defense. Cell 181(3), 674-687.e613. https://doi.org/10.1016/j.cell.2020.03.040.
GSDME	Liu, J., Hong, M., Li, Y., et al. (2022). Programmed Cell Death Tunes Tumor Immunity. Front Immunol 13, 847345. https://doi.org/10.3389/fimmu.2022.847345.
IRF1	Karki, R., Sharma, B.R., Lee, E., et al. (2020). Interferon regulatory factor 1 regulates PANoptosis to prevent colorectal cancer. JCI Insight 5(12). https://doi.org/10.1172/jci.insight.136720.
MAP3K7	Samir, P., Malireddi, R.K.S., and Kanneganti, T.D. (2020). The PANoptosome: A Deadly Protein Complex Driving Pyroptosis, Apoptosis, and Necroptosis (PANoptosis). Front Cell Infect Microbiol 10, 238. https://doi.org/10.3389/fcimb.2020.00238.
MLKL	Zheng, M., Karki, R., Vogel, P., et al. (2020). Caspase-6 Is a Key Regulator of Innate Immunity, Inflammasome Activation, and Host Defense. Cell 181(3), 674-687.e613. https://doi.org/10.1016/j.cell.2020.03.040.
NFS1	Lin, J.F., Hu, P.S., Wang, Y.Y., et al. (2022). Phosphorylated NFS1 weakens oxaliplatin-based chemosensitivity of colorectal cancer by preventing PANoptosis. Signal Transduct Target Ther 7(1), 54. https://doi.org/10.1038/s41392-022-00889-0.
NLRC4	Sundaram, B., and Kanneganti, T.D. (2021). Advances in Understanding Activation and Function of the NLRC4 Inflammasome. Int J Mol Sci 22(3). https://doi.org/10.3390/ijms22031048.
NLRP3	Zheng, M., Karki, R., Vogel, P., et al. (2020). Caspase-6 Is a Key Regulator of Innate Immunity, Inflammasome Activation, and Host Defense. Cell 181(3), 674-687.e613. https://doi.org/10.1016/j.cell.2020.03.040.
PSTPIP2	Samir, P., Malireddi, R.K.S., and Kanneganti, T.D. (2020). The PANoptosome: A Deadly Protein Complex Driving Pyroptosis, Apoptosis, and Necroptosis (PANoptosis). Front Cell Infect Microbiol 10, 238. https://doi.org/10.3389/fcimb.2020.00238.

PYCARD	Zheng, M., Karki, R., Vogel, P., et al. (2020). Caspase-6 Is a Key Regulator of Innate Immunity, Inflammasome Activation, and Host Defense. Cell 181(3), 674-687.e613. https://doi.org/10.1016/j.cell.2020.03.040.
RBCK1	Samir, P., Malireddi, R.K.S., and Kanneganti, T.D. (2020). The PANoptosome: A Deadly Protein Complex Driving Pyroptosis, Apoptosis, and Necroptosis (PANoptosis). Front Cell Infect Microbiol 10, 238. https://doi.org/10.3389/fcimb.2020.00238.
RIPK1	Malireddi, R.K.S., Kesavardhana, S., and Kanneganti, T.D. (2019). ZBP1 and TAK1: Master Regulators of NLRP3 Inflammasome/Pyroptosis, Apoptosis, and Necroptosis (PAN-optosis). Front Cell Infect Microbiol 9, 406. https://doi.org/10.3389/fcimb.2019.00406.
RIPK3	Malireddi, R.K.S., Kesavardhana, S., and Kanneganti, T.D. (2019). ZBP1 and TAK1: Master Regulators of NLRP3 Inflammasome/Pyroptosis, Apoptosis, and Necroptosis (PAN-optosis). Front Cell Infect Microbiol 9, 406. https://doi.org/10.3389/fcimb.2019.00406.
RNF31	Samir, P., Malireddi, R.K.S., and Kanneganti, T.D. (2020). The PANoptosome: A Deadly Protein Complex Driving Pyroptosis, Apoptosis, and Necroptosis (PANoptosis). Front Cell Infect Microbiol 10, 238. https://doi.org/10.3389/fcimb.2020.00238.
TNFAIP3	Samir, P., Malireddi, R.K.S., and Kanneganti, T.D. (2020). The PANoptosome: A Deadly Protein Complex Driving Pyroptosis, Apoptosis, and Necroptosis (PANoptosis). Front Cell Infect Microbiol 10, 238. https://doi.org/10.3389/fcimb.2020.00238.
ZBP1	Malireddi, R.K.S., Kesavardhana, S., and Kanneganti, T.D. (2019). ZBP1 and TAK1: Master Regulators of NLRP3 Inflammasome/Pyroptosis, Apoptosis, and Necroptosis (PAN-optosis). Front Cell Infect Microbiol 9, 406. https://doi.org/10.3389/fcimb.2019.00406.

Supplementary Table 2. Primers and siRNA target sequences.

Name	Sequences
Primers for real-time PCR	Primer:<br>Forward primer CTCCTTCCCACAAGAGCCAC<br>Reverse primer ACTCACTCCGACTGACCTGT
The target sites of siRNA	Si-1:<br>target sequence: atggaaactggccaacattaaaa<br>guide (5′→3′)UUAAUGUUGGCCAGUUUCCAU<br>passenger (5’→3)GGAAACUGGCCAACAUUAAAA<br><br>Si-2:<br>target sequence: cagattaggaaggatgctaaaca<br>guide (5′→3′)UUUAGCAUCCUUCCUAAUCUG<br>passenger (5′→′)GAUUAGGAAGGAUGCUAAACA
